# MICU2, a Paralog of MICU1, Resides within the Mitochondrial Uniporter Complex to Regulate Calcium Handling

**DOI:** 10.1371/journal.pone.0055785

**Published:** 2013-02-07

**Authors:** Molly Plovanich, Roman L. Bogorad, Yasemin Sancak, Kimberli J. Kamer, Laura Strittmatter, Andrew A. Li, Hany S. Girgis, Satya Kuchimanchi, Jack De Groot, Lauren Speciner, Nathan Taneja, Jonathan OShea, Victor Koteliansky, Vamsi K. Mootha

**Affiliations:** 1 Departments of Molecular Biology and Medicine, Massachusetts General Hospital, and Department of Systems Biology, Harvard Medical School, Boston, Massachusetts, United States of America; 2 Broad Institute, Cambridge, Massachusetts, United States of America; 3 Koch Institute for Integrative Cancer Research, Massachusetts Institute of Technology, Cambridge, Massachusetts, United States of America; 4 Alnylam Pharmaceuticals, Inc., Cambridge, Massachusetts, United States of America; University of Texas Health Science Center at San Antonio, United States of America

## Abstract

Mitochondrial calcium uptake is present in nearly all vertebrate tissues and is believed to be critical in shaping calcium signaling, regulating ATP synthesis and controlling cell death. Calcium uptake occurs through a channel called the uniporter that resides in the inner mitochondrial membrane. Recently, we used comparative genomics to identify MICU1 and MCU as the key regulatory and putative pore-forming subunits of this channel, respectively. Using bioinformatics, we now report that the human genome encodes two additional paralogs of MICU1, which we call MICU2 and MICU3, each of which likely arose by gene duplication and exhibits distinct patterns of organ expression. We demonstrate that MICU1 and MICU2 are expressed in HeLa and HEK293T cells, and provide multiple lines of biochemical evidence that MCU, MICU1 and MICU2 reside within a complex and cross-stabilize each other's protein expression in a cell-type dependent manner. Using *in vivo* RNAi technology to silence MICU1, MICU2 or both proteins in mouse liver, we observe an additive impairment in calcium handling without adversely impacting mitochondrial respiration or membrane potential. The results identify MICU2 as a new component of the uniporter complex that may contribute to the tissue-specific regulation of this channel.

## Introduction

The ability of mitochondria to transport calcium is thought to be fundamental for the regulation of cellular bioenergetics [1], [2] and cell death [3], [4]. Mitochondrial calcium uptake and buffering enable these organelles to shape cytosolic calcium transients, resulting in mitochondrial control over key biological processes, including neurotransmission and hormone secretion [5], [6]. The primary route of calcium uptake is via a low affinity calcium “uniporter” located in the inner mitochondrial membrane [7], [8]. This channel mechanism, defined by its dependence on mitochondrial membrane potential, sensitivity to Ru-360 [9] and activation at micromolar calcium concentrations, was first characterized in isolated mitochondria in 1961 [7], [8]. In subsequent decades, the biophysical properties of the mitochondrial calcium uniporter were extensively characterized [10], [11], but its molecular identity remained elusive.

Recently, we coupled observations from comparative physiology with integrative genomics to identify two proteins required for normal mitochondrial calcium handling: MICU1 [12] and MCU [13]. Based on sequence analysis and functional studies, we hypothesized that MCU is the channel-forming subunit of the uniporter, whereas MICU1 fulfilled important criteria for a regulatory protein. This model has been corroborated by independent and complementary studies [14], [15], [16]. Additional studies point to the central role of MICU1 in calcium-mediated insulin signaling [15] and provide evidence that MICU1 may set the calcium threshold for MCU-mediated calcium uptake [16].

For many decades, it has been known that the uniporter exhibits tissue specific regulatory properties [10], [17] whose molecular basis remains to be elucidated. Interestingly, genome sequence analysis reveals that the human genome harbors two additional MICU1 homologs, called EFHA1 and EFHA2, neither of which has been previously studied. Both of these proteins have mitochondrial targeting sequences and were previously identified with high and low confidence, respectively, in MitoCarta, our proteomic characterization of mitochondria from 14 different tissues [18]. Here, we pursue an initial characterization of one of these proteins, EFHA1, which we now re-name MICU2.

## Materials and Methods

### Ethics Statement

All procedures used in animal studies were performed at Alnylam Pharmaceuticals in strict accordance with local and national recommendations and have been approved by the Institutional Animal Care and Use Committee (AAALAC Unit Number 001345, NIH assurance number A4517-01).

### Multiple sequence alignment

Sequences were downloaded from the NCBI protein database. ClustalW2 was used to perform a multiple sequence alignment and generate a phylogenetic tree.

### RNA expression analysis

Data from a publicly available mouse gene expression atlas were downloaded (GEO accession number GSE10246) [19] and summarized using gcRMA normalization [20].

### Cell culture

HeLa and HEK293T cells were received from the ATCC. HeLa cells expressing aequorin targeted to the mitochondrial matrix (mt-AEQ) were purchased from Aequotech (AT-002-H). All cells were grown at 37°C and 5% CO_2_ in Dulbecco's modified Eagle medium (DMEM) (Invitrogen 11995) with 10% FBS (Sigma F6178). HeLa cells expressing mt-AEQ were maintained in 100 µg/ml of geneticin.

### Confocal imaging

HeLa cells were co-transfected with plasmids containing carboxy terminus GFP-tagged MICU2 or Mito-HcRed1 (Clontech 632434). Twenty-four hours after transfection, cells were washed three times with PBS and imaged using a Leica TCS SP5 confocal microscope.

### Quantitative Real-Time PCR

RNA was isolated from HEK293T cells using a Qiagen RNeasy kit and was reverse-transcribed using SuperScript III following the manufacturers' protocols. Quantitative real-time PCR (qPCR) was performed using Taqman assays targeting MICU1 (Hs00381563_m1), MICU2 (Hs0246104_m1) and β-actin (Applied Biosystems Human ACTB #4352935E). Experiments were performed in technical triplicate.

### Immunoprecipitation

Mitochondria were isolated from HEK293T cells that stably express Flag-tagged GFP targeted to mitochondria, MCU-Flag or FLAG-MICU1. 200 µg of protein were solubilized with 200 µl of lysis buffer (50 mM HEPES pH 7.4, 150 mM NaCl, 5 mM EDTA, 0.2% DDM and protease inhibitor tablets (Roche Applied Science 118361170001)) for 10 minutes at 4°C. Lysates were cleared by spinning at 16000 *g* for 10 minutes at 4°C. Cleared lysates were incubated with anti-Flag M2 affinity gel (Sigma A2220) in PBS for 2 hours at 4°C. Immunoprecipitates were washed with 1 ml of lysis buffer 3 times and boiled in 30 µl of sample buffer. One third of the immunoprecipitate was loaded on a 12% SDS-PAGE gel for detection of the indicated proteins. MCU antibody was generated in chicken, MICU1 antibody was generated in rabbit and MICU2 antibody was purchased from Abcam (ab101465). Control antibodies ATP5A (MS507), ATP5B (MS503) and SDHB (MS203) were purchased from MitoSciences.

### Synthesis and selection of siRNA duplexes targeting MICU1 and MICU2

48 and 20 siRNAs were selected for synthesis and screening based on low predicted off-target potentials and 100% homology with mouse sequences NM_144822.2 and NM_028643.3, respectively. Single-strand RNAs were produced at Alnylam Pharmaceuticals as previously described [21]. Hepa-1c1c7 cells seeded at 15,000 cells per well in 96-well plates were transfected with siRNAs using Lipofectamine RNAiMAX according to the manufacturer's protocols. Each experiment was performed in technical duplicate. 18–24 h post-transfection, MICU1 and MICU2 mRNA levels were quantified using a branched-DNA assay (QuantiGene Reagent System, Panomics) according to the manufacturer's protocols. Their mRNA levels were normalized to GAPDH mRNA.

### *In vivo* silencing of MICU1 and MICU2

C57BL/6 mice (Charles River Laboratories) received either PBS or siRNA in lipidoid formulations via weekly tail vein injections as previously described [13], [22], [23], [24]. After overnight fasting, the animals were euthanized by CO_2_ inhalation and the livers were harvested and stored in ice-cold PBS prior to mitochondria isolation. A piece of liver tissue was snap-frozen in liquid nitrogen for mRNA analysis.

### Mitochondrial Isolation

Mitochondria were isolated from mouse liver as previously described [25] and resuspended in a buffer containing 220 mM mannitol, 75 mM sucrose, 10 mM HEPES and 1 mM EDTA adjusted to pH 7.4 with KOH and supplemented with fresh 0.2% BSA prior to use. Mitochondria were stored on ice until further use.

### Measurement of mitochondrial respiration, membrane potential and calcium uptake

Respiration and membrane potential were measured optically as previously described [25]. Values for respiratory control ratios (RCR) and ADP∶O (P∶O) ratios represent the mean +/− s.d. of three independent experiments performed on n = 3 mice. Calcium uptake experiments were performed on a Perkin-Elmer Envision plate reader and Perkin Elmer LS-50B fluorescence spectrometer as previously described [13]. Inset reports linear fits between 5 and 10 s from experiments performed on n = 3 mice.

### RNA interference

To silence MICU1, we used short-hairpin RNA (shRNA) constructs TRCN0000053370 (5′-GCAATGGCGAACTGAGCAATA-3′, shMICU1_a_) and TRCN0000053368 (5′-GCAGCTCAAGAAGCACTTCAA-3′, shMICU1_b_) from the Broad Institute's RNAi Consortium (TRC) previously validated in mt-AEQ HeLa cells. To silence MICU2, we used shRNA constructs TRCN0000055848 (5′-GCCATGCAGATGTTCAGTTTA-3′, shMICU2_a_) and TRCN0000055850 (GCTGCAGAAGATCATAAGTAA, shMICU2_b_). As controls, shRNAs targeting GFP, RFP and LACZ were used. All shRNAs were provided in a lentiviral vector (pLKO.1) through the TRC. Lentiviral production and infection were performed as previously described [26]. Cells were selected 24 hours after infection with 2 µg/ml of puromycin.

### cDNA rescue experiments

A lentiviral vector (pLEX983) for expressing C-terminal V5-tagged cDNA was obtained from the TRC. Full-length MICU2 cDNA was synthesized (Blue Heron Biotechnology) and cloned into pLEX983. Lentivirus was produced using cDNAs encoding GFP, MICU2 or MICU1 resistant to TRCN0000053370 as previously described [26]. Virus was used to infect HeLa cells stably expressing mt-AEQ. Cells were selected 24 hours after infection with 5 µg/ml of blasticidin.

### Measurement of mitochondrial calcium in HeLa cells expressing mt-AEQ

40,000 cells were seeded in a 96-well plate and incubated overnight. Mitochondrial calcium was measured following histamine treatment as previously described [27]. Light emission was measured at 469 nm every 0.1 s using a luminometer (MicroBeta2 LumiJET Microplate Counter PerkinElmer). Luminescence was normalized to account for cell number.

### Blue native PAGE studies

5 µg of mitochondria isolated from mouse liver or HeLa cells were solubilized in 2% digitonin on ice for 30 minutes. Electrophoresis was performed using the Novex NativePAGE Bis-Tris Gel System from Invitrogen. Western blot analysis was performed using an MCU antibody generated in chicken. A commercially available antibody to ATP5A (MitoSciences MS507) was used as a loading control.

## Results

### MICU1, MICU2 and MICU3 form a family of paralogous genes

We previously used comparative genomics to identify MICU1, a mitochondrial protein essential for mitochondrial calcium handling in HeLa cells [12]. MICU1 (previously known as CBARA1 or EFHA3) has a mitochondrial targeting sequence as well as two evolutionarily conserved EF hands. Sequence analysis reveals that MICU1 shares approximately 25% sequence identity with two human genes, *EFHA1* and *EFHA2*, which have not been studied before. These three proteins are conserved in vertebrates, whereas only one homolog is present in plants and protozoa.

**Figure 1:**
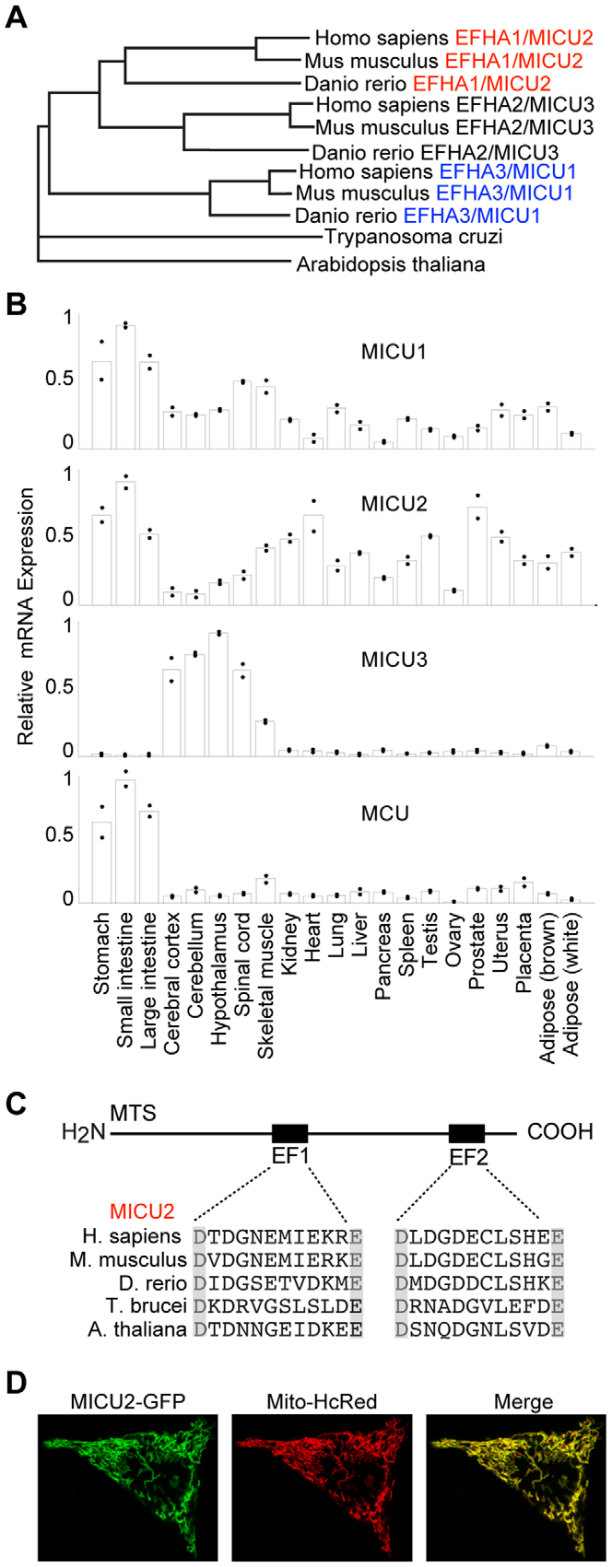
MICU2 is paralogous to MICU1 and localizes to mitochondria. A. MICU1, MICU2 and MICU3 share a common ancestor and are present in multiple vertebrate species. B. RNA expression analysis of MICU1, MICU2, MICU3 and MCU across 21 mouse tissues. For each tissue, the dots represent individual replicate measures and the bars represent mean values. C. MICU2 has two evolutionarily conserved EF hands. D. Representative confocal images of HeLa cells cotransfected with MICU2-GFP and Mito-HcRed1.

To elucidate the evolutionary relationship of these three proteins, we performed a multiple sequence alignment using homologs from vertebrates, plants and protozoa. This analysis placed the protozoa and plant homologs as outgroups on a phylogenetic tree (Fig. 1a), indicating that EFHA1 and EFHA2 are vertebrate paralogs of MICU1 that likely arose from a gene duplication event prior to vertebrate evolution. We now re-name EFHA1 as MICU2 and EFHA2 as MICU3.

Like MICU1, MICU2 and MICU3 have N-terminal targeting sequences consistent with mitochondrial localization [28]. In our previous proteomic studies, MICU2 and MICU3 were detected in mitochondria from various mouse tissues, although their expression patterns differed significantly [18]. MICU1 was broadly expressed and detected in 12 out of 14 mouse tissues, whereas MICU2 was detected in 7 out of 14 tissues with strong expression in visceral organs. In contrast, MICU3 was found in 6 out of 14 tissues with a strong signature in skeletal muscle and the central nervous system. Despite being detected in multiple mouse tissues, MICU3 lacked other evidence of being mitochondrial, scoring just below the stringent threshold required to be a part of the MitoCarta collection. This is in contrast to MICU2, which was reported in MitoCarta and localized to the mitochondria using high-content microscopy to visualize its subcellular localization.

Analysis of published microarrays of MICU1-3 across 21 different mouse tissues revealed a similar expression pattern as predicted by MitoCarta (Fig. 1b). MICU2 was strongly expressed in visceral organs whereas MICU3 was expressed almost exclusively in neural tissues and skeletal muscle (Fig. 1b). In the present study, we focus on MICU2 due to greater expression in visceral organs in which we can apply *in vivo* siRNA technology to achieve silencing.

Sequence comparison of human MICU1 and MICU2 reveals highly conserved domain architecture (Fig. 1c). Similar to MICU1, MICU2 has two EF-hands separated by a long stretch of residues predicted to form α-helices. In previous work, we showed that the calcium-coordinating residues in the EF-hands of MICU1 are highly conserved across species [12]. Likewise, MICU2 demonstrates perfect conservation in the acidic residues that cap the EF-hands across evolution (Fig. 1c).

### MICU2-GFP localizes exclusively to mitochondria

We began by confirming that MICU2-GFP localizes exclusively to mitochondria with confocal microscopy, which showed that a carboxy terminus GFP-tagged MICU2 overlapped with mitochondrial marker Mito-HcRed1 (Fig. 1d).

### MICU1 and MICU2 stabilize each other's protein expression

**Figure 2:**
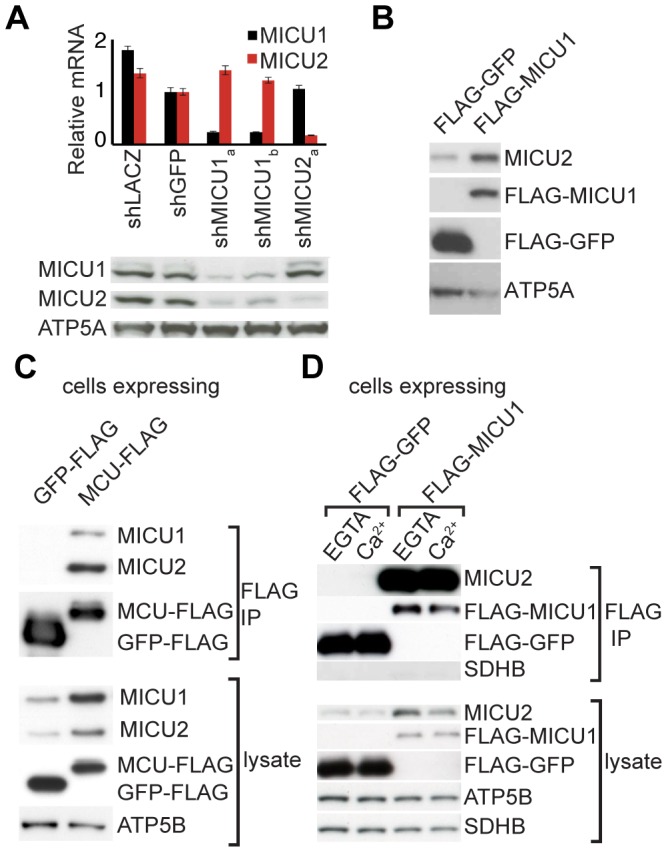
MICU1 and MICU2 stabilize each other's expression and interact with MCU. A. Whole cell lysates from HEK293T cells stably expressing a control shRNA (shGFP and shLACZ) or a shRNA targeting MICU1 (shMICU1_a_ and shMICU1_b_) or MICU2 (shMICU2_a_) were analyzed using qPCR and western blot. The relative mRNA is reported using β-actin as an endogenous control and normalized to shGFP for each target. Whole cell lysates were blotted with anti-MICU1, anti-MICU2 and control anti-ATP5A. B. Whole cell lysates from HEK293T cells stably expressing FLAG-GFP or FLAG-MICU1 were lysed and blotted with anti-MICU2, anti-FLAG and control anti-ATP5A. C–D. Mitochondria isolated from HEK293T cells stably expressing MCU-FLAG (C) or FLAG-MICU1 (D) were solubilized with 0.2% DDM and subjected to anti-FLAG immunoprecipitation. Immunoprecipitates and lysate were blotted with anti-FLAG, anti-MICU1, anti-MICU2 and control anti-ATP5B and anti-SDHB.

In previous studies, we demonstrated that MICU1 regulates mitochondrial calcium handling in HeLa cells [12]. Since MICU1 and MICU2 are paralogs with conserved domain architecture, it is possible that they have redundant roles and are expressed in a mutually exclusive manner across different cell types. To address this possibility, we blotted whole cell lysate from HEK293T and HeLa cells for MICS1U1 and MICU2, which revealed expression of both proteins (Fig. 2a, S1, S2a). Interestingly, in the setting of MICU1 knockdown, there was a significant reduction in MICU2 protein expression in both HEK293T and HeLa cells despite preserved MICU2 RNA levels (Fig. 2a, S2a). This phenomenon was observed following treatment with multiple hairpins (shMICU1_a_ and shMICU1_b_), indicating that this result is a biological consequence of MICU1 loss rather than an off target effect. Conversely, overexpression of MICU1 in HEK293T resulted in higher levels of MICU2 (Fig. 2b). Interestingly, in the setting of MICU2 knockdown, a reduction of MICU1 was observed in HeLa cells (Fig. S2a) whereas no effect on MICU1 expression was observed in HEK293T cells (Fig. 2a). This discrepancy may reflect additional mechanisms contributing to MICU1 stability in HEK293T cells. Collectively, these studies demonstrate that MICU1 and MICU2 are expressed in multiple cell types and that their protein expression is contingent on one another.

### MICU2 associates with the MICU1/MCU complex

In our previous studies, we demonstrated that MICU1 and MCU physically interact either directly or indirectly [13]. To determine whether MICU2 physically interacts with these proteins, we performed immunoprecipitation on cells stably expressing either MCU-FLAG or GFP-FLAG. Although endogenous MICU1 and MICU2 were present in cell lysate from both cell lines, immunoprecipitation with an anti-FLAG antibody specifically recovered MICU1 and MICU2 from the MCU-FLAG cell line (Fig. 2c). It is notable that the heterologous expression of MCU-FLAG resulted in increased protein expression of both MICU1 and MICU2 as seen in the whole cell lysate (Fig. 2c), suggesting that MCU overexpression stabilizes the protein expression of MICU1 and MICU2. In addition, immunoprecipitation performed on cells stably expressing either MICU1-FLAG or GFP-FLAG demonstrated specific recovery of MICU2 with MICU1 in the presence of excess calcium or EGTA (Fig. 2d). Collectively, these results (Fig. 2a–d) strongly support the notion that MICU1, MICU2 and MCU physically interact to form a complex.

### MICU1 and MICU2 can be silenced in vivo in mouse liver using siRNA technology

Our sequence analysis, expression analysis and biochemical data motivated us to pursue studies in which we could directly assess the contribution of MICU2 to mitochondrial calcium uptake. To evaluate the impact of MICU2 loss on mitochondrial calcium uptake, we performed *in vivo* silencing of MICU2 in mouse liver using technology developed by Alnylam Pharmaceuticals [13]. Since a key question was whether MICU1 and MICU2 contribute independently to mitochondrial calcium uptake, we also performed *in vivo* silencing of MICU1 in isolation and in combination with MICU2.

**Figure 3:**
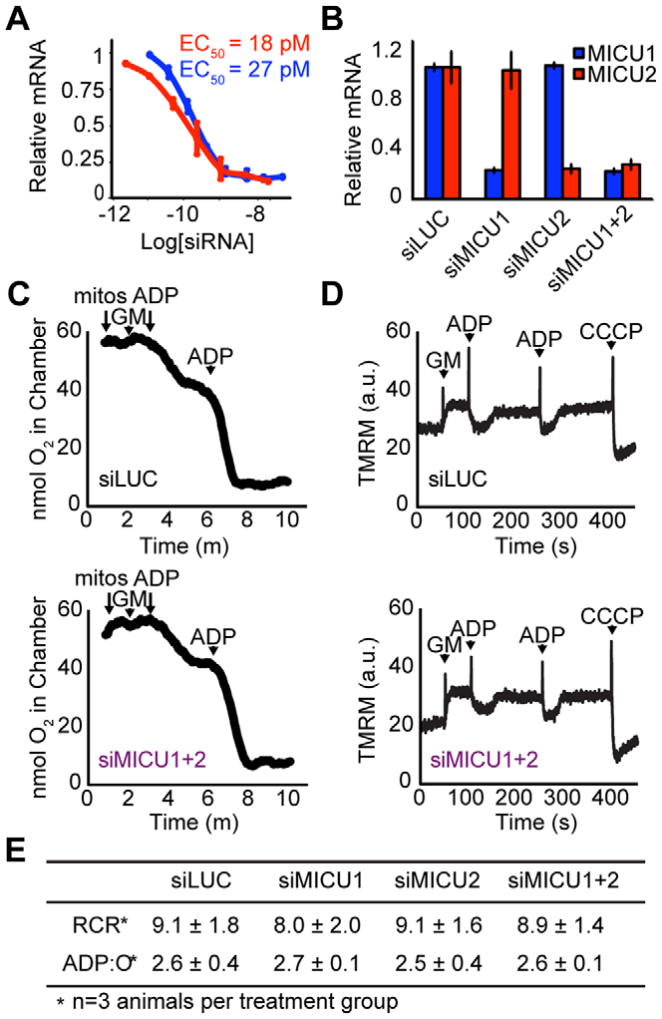
MICU1 and MICU2 can be silenced *in vivo* in mouse liver using siRNA technology. A. *In vitro* dose-response curves of selected duplexes targeting MICU1 and MICU2. B. Relative expression of MICU1 and MICU2 mRNA after 6 weekly injections normalized to siLUC mice. C. Representative oxygen consumption traces measured in isolated mitochondria from siLUC (top) and siMICU1+2 (bottom) mice. Arrows denote addition of mitochondria, glutamate and malate (G/M), ADP and uncoupler (carbonyl cyanide m-chlorophenylhydrazone, CCCP). Respiratory control ratios (RCR) and ADP∶O ratios (P∶O) were calculated from experiments performed on three separate mice per group. D. Representative mitochondrial membrane potential traces measured in isolated mitochondria from siLUC (top) and siMICU1+2 (bottom) mice using tetramethyl rhodamine methyl ester (TMRM). E. Respiratory control ratios (RCR) and ADP∶O ratios (P∶O) were comparable among all treatment groups.

We screened siRNA duplexes for both genes using previously described siRNA design and delivery technology [13]. Duplexes were transfected at concentrations from 1.25 to 5 nM, and the 3 siRNAs conferring the best knockdown were tested at different concentrations in the range of 7pM to 5 nM to estimate their EC_50_ in a mouse liver cell line (Fig. 3a). As a negative control, we used a siRNA duplex that targets firefly luciferase (siLUC). We performed a large-scale synthesis of one siRNA duplex and encapsulated it into a lipid-based formulation optimized for liver-specific delivery. Although mRNA knockdown was achieved at 24 hours, weekly tail vein injections of the siRNA duplexes were carried out over a six-week period to achieve *in vivo* knockdown of MICU1 and MICU2 (Fig. 3b). Similar to *in vivo* silencing of MCU [13], mice did not exhibit any signs of distress. Their weight was stable, and the gross appearance of their livers did not differ.

### Mitochondrial membrane potential and respiration are intact following *in vivo* silencing of MICU1/MICU2 in mouse liver

We evaluated the impact of silencing MICU1 and MICU2 on mitochondrial respiration and membrane potential (*Ψ_m_*) to ensure that silencing did not cause respiratory chain collapse leading to a secondary defect in calcium uptake. Mitochondria isolated from control and knockdown livers underwent robust respiratory transitions as demonstrated by their comparable respiratory control ratios (RCR) (Fig. 3c, e). In addition, comparable ADP∶O ratios indicate intact ATP-coupled mitochondrial respiration. Together, these results indicate that silencing MICU1 or MICU2 did not alter baseline mitochondrial respiration or oxidative phosphorylation. In mitochondria isolated from all treatment groups, membrane potential was responsive to ADP and fully depolarized by the uncoupler carbonyl cyanide *m*-chlorophenylhydrazone (CCCP), indicating that silencing MICU1 or MICU2 did not abolish the mitochondrial membrane potential (Fig. 3d). It is notable that these findings are similar to what we previously reported for *in vivo* silencing of MCU in mouse liver [13].

### *In vivo* silencing of MICU1 and MICU2 in mouse liver results in altered mitochondrial calcium handling

**Figure 4:**
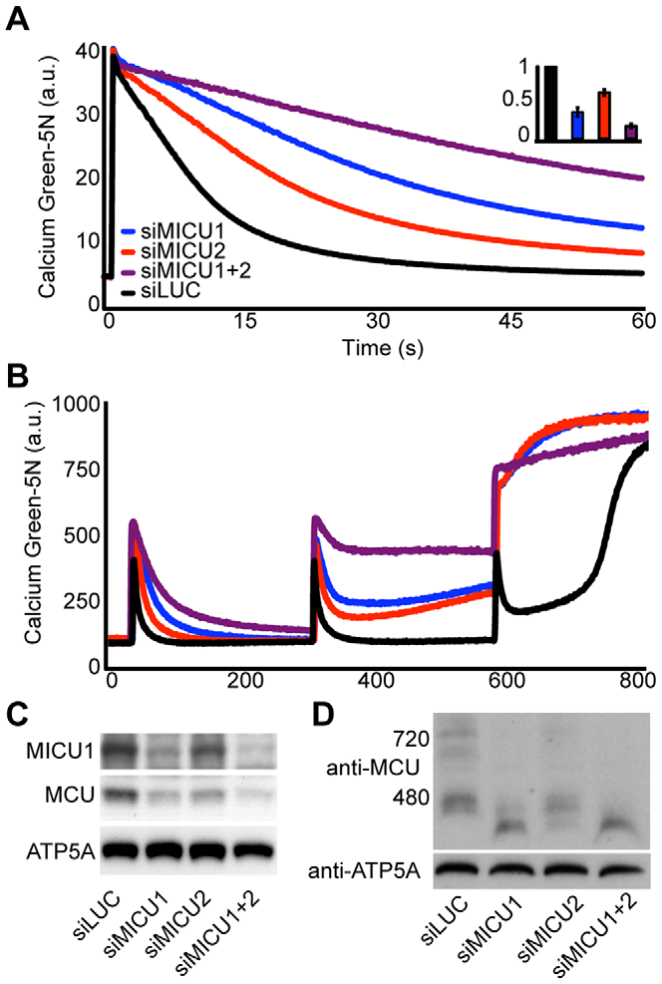
Silencing MICU1 and MICU2 results in impaired calcium handling and alters MCU complex size in mouse liver. A. Calcium uptake in energized liver mitochondria following the addition of 50 µM CaCl_2_. Inset reports linear fits of uptake between 5 and 10 s normalized to siLUC uptake. B. Calcium uptake in energized liver mitochondria following the addition of multiple spikes of 50 µM CaCl_2_. C. Mouse liver mitochondria isolated from animals treated with siLUC, siMICU1, siMICU2 or siMICU1+2 were blotted with anti-MICU1, anti-MCU and control anti-ATP5A. D. BN-PAGE analysis of mouse liver mitochondria isolated from animals treated with siLUC, siMICU1, siMICU2 or siMICU1+2. Protein was transferred to a membrane and blotted with anti-MCU and control anti-ATP5A.

Next, we analyzed calcium uptake kinetics in mitochondria isolated from control and knockdown mice. We added a single, large 50 µM spike of calcium to mitochondria suspended in buffer containing extramitochondrial Calcium Green-5N (CG5N) and observed the rate of calcium clearance by measuring CG5N fluorescence. Mitochondria from siMICU1 and siMICU2 mice demonstrated moderately impaired calcium uptake kinetics whereas mitochondria from siMICU1+siMICU2 mice demonstrated an additive defect (Fig. 4a). This result was also observed when multiple spikes of calcium were added to mitochondria (Fig. 4b). Interestingly, knockdown mitochondria were unable to fully buffer a second pulse of calcium, whereas control mitochondria rapidly buffered the entire pulse (Fig. 4b). Knockdown mitochondria demonstrated premature release of calcium, suggesting that knockdown mitochondria were sensitized to release calcium (Fig. 4b). In addition, MCU protein expression was significantly decreased in knockdown mitochondria (Fig. 4c). The degree of MCU protein loss correlated with the phenotype strength, raising the question of whether MCU loss was responsible for at least part of the observed phenotype. Similar to the effect of MICU2 knockdown in HeLa cells, loss of MICU2 in mouse liver resulted in decreased expression of MICU1 to about half of wild-type levels (densitometry not shown). By extending the phenomenon of cross-stabilization to mouse tissues, this result strongly suggests that a fundamental role of these paralogs is stabilizing each other's protein expression.

### *In vivo* silencing of MICU1 and MICU2 alters MCU complex size

In previous studies, we demonstrated that MCU is present in a complex of approximately 480 kDa using blue native polyacrylamide gel electrophoresis (BN-PAGE) and western blot (WB) analysis [13]. To gain insight into how silencing these proteins may affect the uniporter complex, we solubilized control and knockdown mitochondria in digitonin and performed BN-PAGE and western blot analysis, which revealed decreased levels of MCU complex in knockdown mitochondria (Fig. 4d). In addition, blotting with anti-MCU revealed a shift in MCU complex size in knockdown mitochondria relative to siLUC (Fig. 4d). The percentage of shifted MCU complex was greater in si-MICU1 mitochondria compared to si-MICU2 mitochondria. The basis for this difference is unclear, but it may reflect less efficient MICU2 knockdown or increased sensitivity of the uniporter complex to MICU1 loss relative to MICU2 loss. Interestingly, the degree of shift correlated with the severity of calcium uptake. This result was also observed in human cell lines stably expressing shMICU1_a_ and shLZ (Fig. S2b). The shift in the MCU complex size could be rescued by introducing a cDNA encoding MICU1 that was resistant to shRNA degradation, suggesting that this shift was specific to MICU1 knockdown rather than an off-target effect (data not shown).

### Redundant or complementary roles of MICU1 and MICU2?

An important question is whether MICU1 and MICU2 have redundant or complementary roles in mitochondrial calcium uptake. The fact that MICU1 and MICU2 are both expressed in multiple human cell lines and mouse liver and that simultaneous silencing results in an additive calcium handling defect suggests that they contribute independently to mitochondrial calcium handling. However, the finding that their loss produces similar defects, including reduced calcium uptake kinetics, premature calcium release and a shift in the MCU complex size, indicates that they may have overlapping molecular roles in mitochondrial calcium handling.

To explore this question, we used a commercially available mt-AeQ HeLa reporter cell line that reports matrix free calcium. In this background, we stably expressed a shRNA targeting MICU1, which resulted in a severely diminished rise in free matrix calcium following histamine stimulation as we previously reported [12]. Subsequently, we introduced a cDNA encoding either MICU1 (with synonymous mutations at the shRNA sites) or MICU2. As expected, MICU1 robustly restored the rise in matrix calcium following histamine stimulation (Fig. S2c). Although expression of MICU2 rescued the calcium phenotype, it also restored MICU1 protein levels to approximately half of wild-type levels (Fig. S2c, d). This result precluded a definitive evaluation of whether MICU2 could compensate for MICU1 because it could be explained by multiple mechanisms, including MICU2 stabilizing low levels of remaining MICU1 or directly replacing MICU1 as a functional paralog. In contrast to mitochondria isolated from mouse liver, it is important to point out that MICU1 knockdown does not result in decreased MCU expression in cultured HeLa cells (Fig. S2d), which may reflect cell-type differential regulation.

## Discussion

Mitochondrial calcium transport is a highly conserved phenomenon that is linked to a diverse set of cellular processes, including cellular metabolism [2], [5] and programmed cell death [4], [26]. Given the lack of cell-permeant uniporter inhibitors and the previously unknown molecular identity of the uniporter, the functional studies to date have been largely correlative. The discovery of MICU1 and MCU [12], [13] opens the door to an endless set of targeted genetic and biochemical studies that will enable a detailed molecular understanding of mitochondrial calcium uptake and the mechanisms that govern its regulation.

MICU1 is a part of a duplicated gene family found in all major branches of life, including metazoans, plants, protozoa and fungi, with lineage specific losses [29]. In the current paper, we show that MICU1, MICU2 and MICU3 are conserved in vertebrates and that they exhibit distinct patterns of expression (Fig. 1b), suggesting complementary roles in controlling uniporter physiology. Based on MitoCarta, MICU2 is a high confidence mitochondrial-localized protein whereas MICU3 is likely mitochondrial but with lower confidence [18]. We prioritized MICU2 for functional studies, but MICU3 likely has a role in mitochondrial calcium handling in a subset of tissues, notably in the CNS and skeletal muscle, though this remains to be formally proven. Although we showed in previous work that loss of MICU1 in HeLa cells results in impaired mitochondrial calcium handling [12], it was unclear if this property extended to other cell types. Our current study extends this finding to mouse liver, establishing the central role of MICU1 in calcium handling in mammalian mitochondria.

Our biochemical and genetic studies strongly support the notion that MCU, MICU1 and MICU2 reside within a complex. This idea is supported by co-IP experiments as well as by the novel observation of cross-stabilization of these proteins. This was most evident in HEK293T cells in which we performed the majority of our biochemical experiments. Silencing MICU1 resulted in loss of MICU2 protein (Fig. 2c), and forced expression of either MICU1 or MCU led to apparent stabilization of MICU1 and MICU2 protein (Fig. 2b, c). Similar effects are observed in HeLa cells, where MICU1 protein levels were also sensitive to MICU2 knockdown (Fig. S2a). In mouse liver, we observed that silencing MICU1 or MICU2 led to a shift in the large molecular weight MCU complex (Fig. 4d) previously described [13]. Together, these studies reveal that MICU2 is a genuine member of the uniporter complex.

At present, the precise molecular function of MICU1, MICU2 and MICU3 remain unclear. In this study, we performed *in vivo* silencing of MICU1 and MICU2, which resulted in reduced mitochondrial calcium clearance in response to large 50 µM calcium pulses. Given the reconstitution data suggesting that MCU is the pore-forming subunit of the uniporter, possible roles for MICU2 could include: (i) Ca2+ sensing and regulation of MCU, (ii) calcium buffering with a secondary impact on transport or (iii) assembly and stabilization of MCU. A recent study provides compelling evidence that MICU1 sets the threshold for mitochondrial calcium uptake without affecting the kinetics properties of the pore [16]. How MICU2 contributes to this mechanism remains an important outstanding question.

Although our results show that MICU1 and MICU2 play complementary roles at a physiological level, it remains unclear whether they have distinct or redundant molecular functions. The evolutionary conservation of MICU1, MICU2 and MICU3 in vertebrates, their distinct patterns of expression across organs and the presence of MICU1 and MICU2 in two different cell types indicate complementary roles in cellular physiology. However, it is unclear if they are redundant on a molecular level. We attempted to complement a strong MICU1 phenotype in HeLa cells that we previously reported by expressing MICU2 on a MICU1 knockdown background. Although MICU2 was able to rescue this phenotype, we found that MICU2 also stabilized the protein expression of the small amount of MICU1 (Fig. S2d), confounding the interpretation. Resolving this question will require the use of a null genetic background in which the activity of one paralog can be rigorously assessed in the absence of the other paralog.

Our biochemical findings have important implications for the interpretation of functional studies of the uniporter that employ genetic silencing or overexpression. The current study demonstrates that when uniporter protein components are genetically silenced, this perturbation may impact the protein stability of companion proteins, and that cross-stabilization may represent a cell-type specific phenomenon. For example, forced expression of MCU has been reported to give a gain of function phenotype, yet we observe that forced expression of MCU also leads to elevated levels of MICU1 and MICU2 in HEK293T cells (Fig. 2c). Silencing of MICU1 and MICU2, either alone or in combination, in mouse liver appears to have an impact on the abundance of MCU protein levels (Fig. 4c), which likely contributed to decreased mitochondrial calcium clearance in these assays (Fig. 4a, b). Cross-stabilization is not unusual in large, protein complexes, including those of the respiratory chain, where the expression of subunits is nucleated and stabilized by other partners. Future studies of the uniporter need to be cognizant of the ability of MCU, MICU1, and MICU2 to impact each other's protein expression, as genetic silencing studies may misattribute a molecular function to the target protein when indeed the impact may be indirect. In addition, it will be important to consider other mechanisms, including those involving MCUR1 [30], LETM1 [31] and NCLX [32] that may additionally influence mitochondrial calcium physiology.

It is tempting to speculate that the relative expression of MICU1, MICU2 and MICU3 differ in a cell and state-specific manner to regulate mitochondrial calcium handling. Under this model, multiple paralogs could be constitutively expressed in a single cell type, but variation in their relative abundance could give rise to functional differences in mitochondrial calcium handling, as has been previously documented across tissues [10], [17]. If this model proves to be correct, it may open up the possibility of therapeutically targeting the uniporter in a tissue specific manner.

## Supporting Information

Figure S1 **Antibodies for MICU1 and MICU2 do not cross-react.** Whole cell lysates from HEK293T cells stably expressing a control shRNA (shLZ) or shRNA targeting MICU1 (shMICU1_a_) or MICU2 (shMICU2_a_) were blotted with anti-MICU1, anti-MICU2 and control anti-ATP5A.
(TIF)

Figure S2 **Analysis of MICU1, MICU2 and MCU in HeLa cells.** A. Whole cell lysates from HeLa cells stably expressing a control shRNA (shGFP and shLACZ) or a shRNA targeting MICU1 (shMICU1_a_ and shMICU1_b_) or MICU2 (shMICU2_a_ and shMICU2_b_) were blotted with anti-MICU1, anti-MICU2 and control anti-ATP5A. B. BN-PAGE analysis of mitochondria isolated from HeLa cells stably expressing shGFP or shMICU1_a_. Protein was transferred to a membrane and blotted with anti-MCU and control anti-ATP5A. C. Luminescence measurements of mitochondrial matrix calcium following histamine stimulation in HeLa cells stably expressing aequorin targeted to the mitochondrial matrix (mean ± s.e.m., *n* = 4). Inset reports statistics on the maximal luminescence (mean ± s.d., n = 8, *P<0.001). D. Western blot analysis of cells stably expressing shGFP and GFP, shMICU1_a_ and GFP, shMICU1_a_ and MICU1-V5 or shMICU1_a_ and MICU2-V5.
(TIF)
