# Correction: MICU2, a Paralog of MICU1, Resides within the Mitochondrial Uniporter Complex to Regulate Calcium Handling

**DOI:** 10.1371/journal.pone.0352309

**Published:** 2026-06-24

**Authors:** Molly Plovanich, Roman L. Bogorad, Yasemin Sancak, Kimberli J. Kamer, Laura Strittmatter, Andrew A. Li, Hany S. Girgis, Satya Kuchimanchi, Jack De Groot, Lauren Speciner, Nathan Taneja, Jonathan OShea, Victor Koteliansky, Vamsi K. Mootha

Following publication of this article [1], an error was identified in Fig 2. Specifically, the SDHB lysate panel in Fig 2D is incorrect and is a duplicate of the ATP5B panel in Fig 2D. With this Correction, the authors provide an updated Fig 2 with the correct SDHB lysate panel from the original experiment and a revised Fig 2B, updated to present FLAG-MICU1 and FLAG-GFP panels in their original blot form as one panel.

The authors stated that the error likely happened in the preparation of the final versions of the revised manuscript and confirmed that the lysate and immunoprecipitate (IP) samples were run on the same gel. Due to a strong signal in the FLAG IP lanes, a shorter exposure was used for the FLAG-MICU1 and FLAG-GFP panels compared with the corresponding lysate panels, and both the short and long exposures of this underlying immunoblot can be viewed in S1 File.

A member of the *PLOS One* Editorial Board was consulted and advised that the specificity of the antibodies used for Fig 2D supports the reliability of the results.

With this Correction, the authors provide the original image data underlying Fig 2D in S1 File and they confirmed that all other image and quantitative data underlying the rest of the results are available.

**Fig 2:**
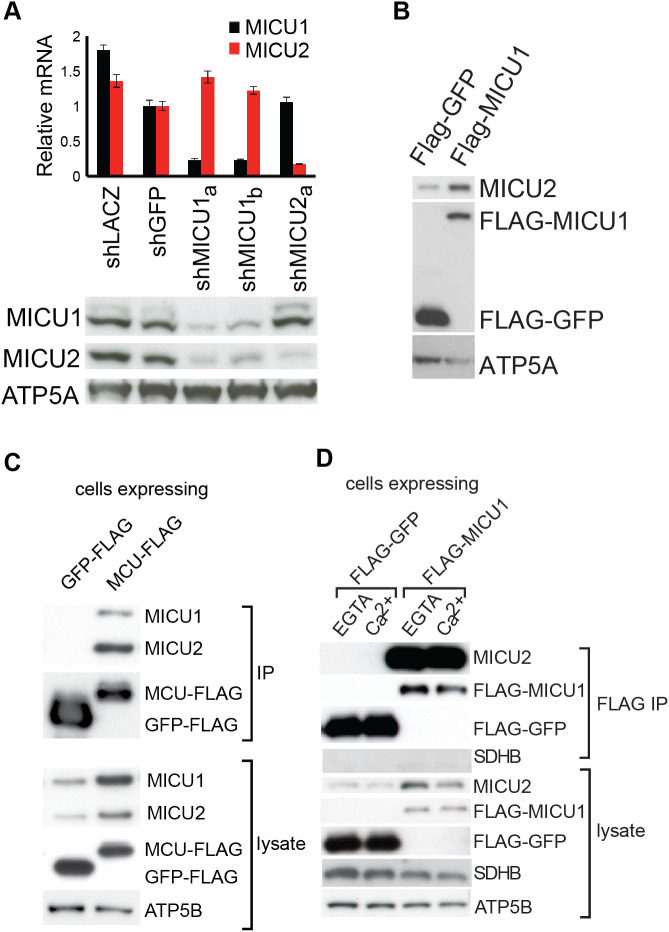
MICU1 and MICU2 stabilize each other's expression and interact with MCU. **A.** Whole cell lysates from HEK293T cells stably expressing a control shRNA (shGFP and shLACZ) or a shRNA targeting MICU1 (shMICU1a and shMICU1b) or MICU2 (shMICU2a) were analyzed using qPCR and western blot. The relative mRNA is reported using β-actin as an endogenous control and normalized to shGFP for each target. Whole cell lysates were blotted with anti-MICU1, anti-MICU2 and control anti-ATP5A. **B.** Whole cell lysates from HEK293T cells stably expressing FLAG-GFP or FLAG-MICU1 were lysed and blotted with anti-MICU2, anti-FLAG and control anti-ATP5A. **C–D**. Mitochondria isolated from HEK293T cells stably expressing MCU-FLAG (C) or FLAG-MICU1 (D) were solubilized with 0.2% DDM and subjected to anti-FLAG immunoprecipitation. Immunoprecipitates and lysate were blotted with anti-FLAG, anti-MICU1, anti-MICU2 and control anti-ATP5B and anti-SDHB.

## Supporting information

S1 File Image data underlying Fig 2D. Annotated scans for all panels in Fig 2D.
(PDF)
