# Supplementary figures and images for: MICU2, a Paralog of MICU1, Resides within the Mitochondrial Uniporter Complex to Regulate Calcium Handling
Source: PLoS One. 2013 Feb 7;8(2):e55785. doi: 10.1371/journal.pone.0055785 (PMC3567112; doi:10.1371/journal.pone.0055785)

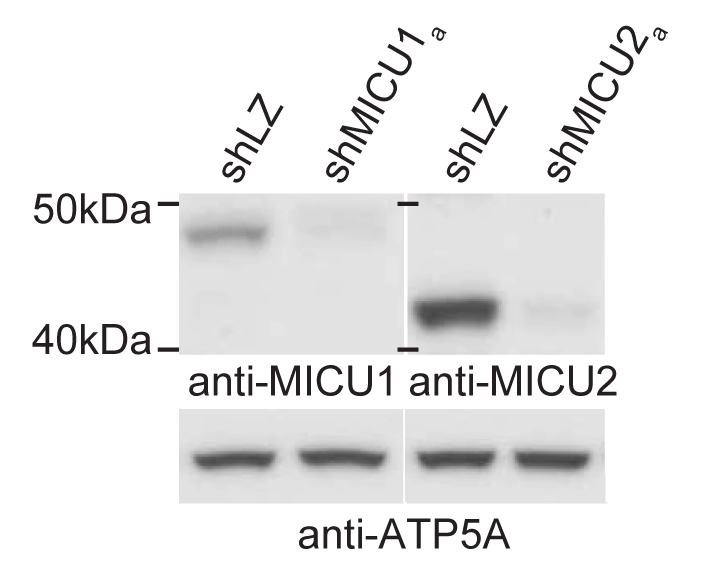

Supplement: Figure S1 — Antibodies for MICU1 and MICU2 do not cross-react. Whole cell lysates from HEK293T cells stably expressing a control shRNA (shLZ) or shRNA targeting MICU1 (shMICU1a) or MICU2 (shMICU2a) were blotted with anti-MICU1, anti-MICU2 and control anti-ATP5A. (TIF) [file pone.0055785.s001.tif]

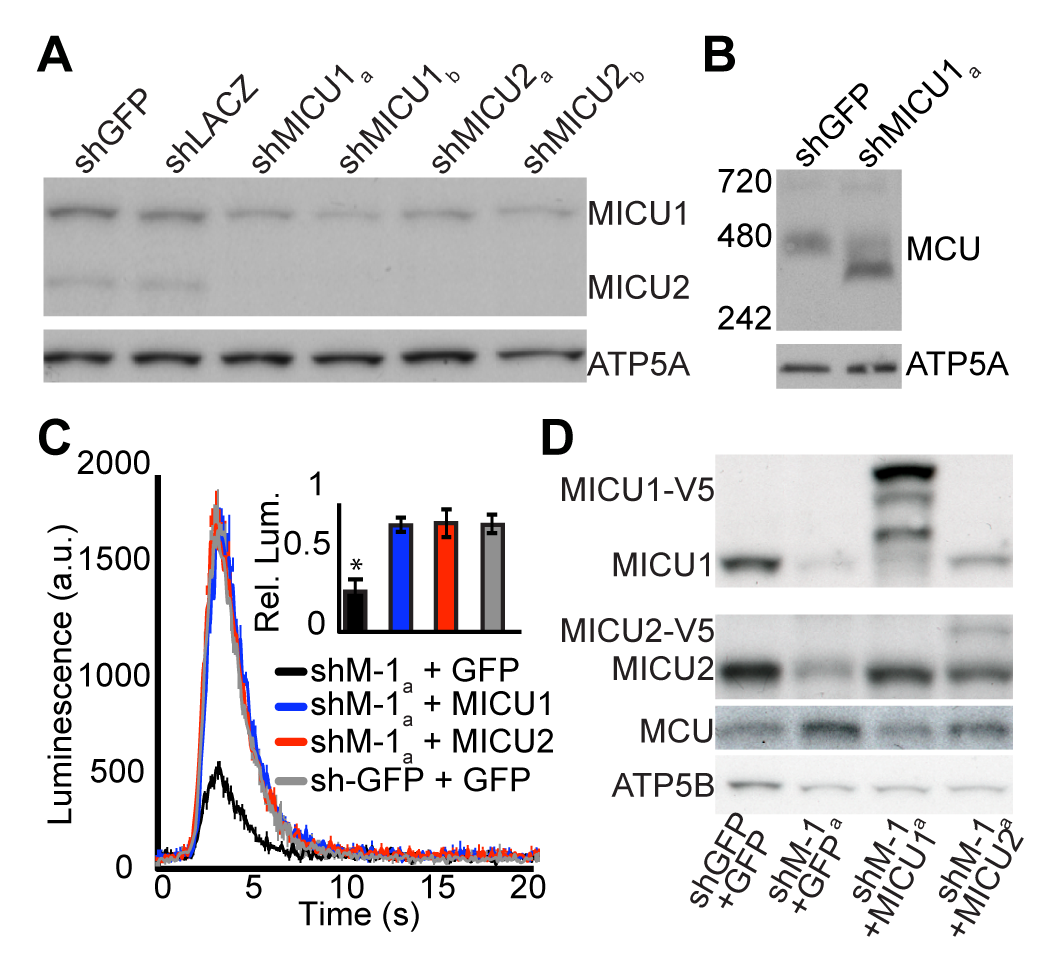

Supplement: Figure S2 — Analysis of MICU1, MICU2 and MCU in HeLa cells. A. Whole cell lysates from HeLa cells stably expressing a control shRNA (shGFP and shLACZ) or a shRNA targeting MICU1 (shMICU1a and shMICU1b) or MICU2 (shMICU2a and shMICU2b) were blotted with anti-MICU1, anti-MICU2 and control anti-ATP5A. B. BN-PAGE analysis of mitochondria isolated from HeLa cells stably expressing shGFP or shMICU1a. Protein was transferred to a membrane and blotted with anti-MCU and control anti-ATP5A. C. Luminescence measurements of mitochondrial matrix calcium following histamine stimulation in HeLa cells stably expressing aequorin targeted to the mitochondrial matrix (mean ± s.e.m., n = 4). Inset reports statistics on the maximal luminescence (mean ± s.d., n = 8, *P<0.001). D. Western blot analysis of cells stably expressing shGFP and GFP, shMICU1a and GFP, shMICU1a and MICU1-V5 or shMICU1a and MICU2-V5. (TIF) [file pone.0055785.s002.tif]
