# Supplementary figures and images for: Correction: MICU2, a Paralog of MICU1, Resides within the Mitochondrial Uniporter Complex to Regulate Calcium Handling
Source: PLoS One. 2026 Jun 24;21(6):e0352309. doi: 10.1371/journal.pone.0352309 (PMC13293399; doi:10.1371/journal.pone.0352309)

Published Figure 2D

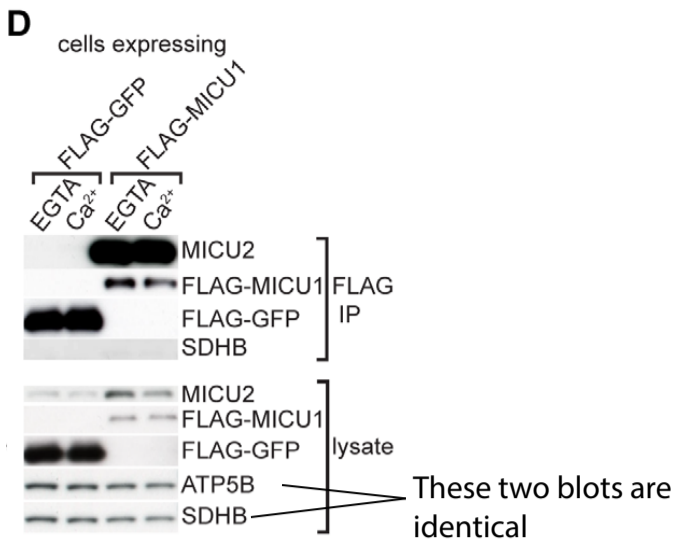

Corrected Figure 2D

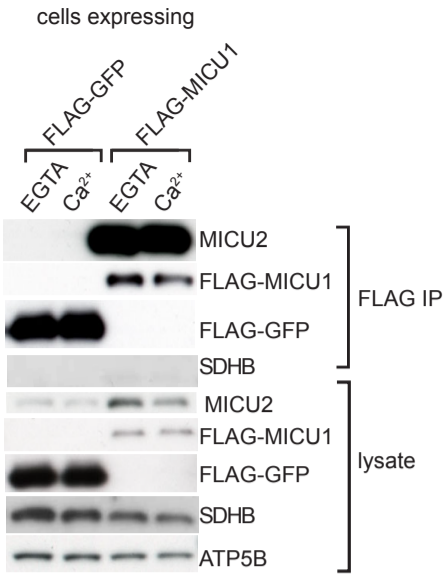

Corrected Figure 2D

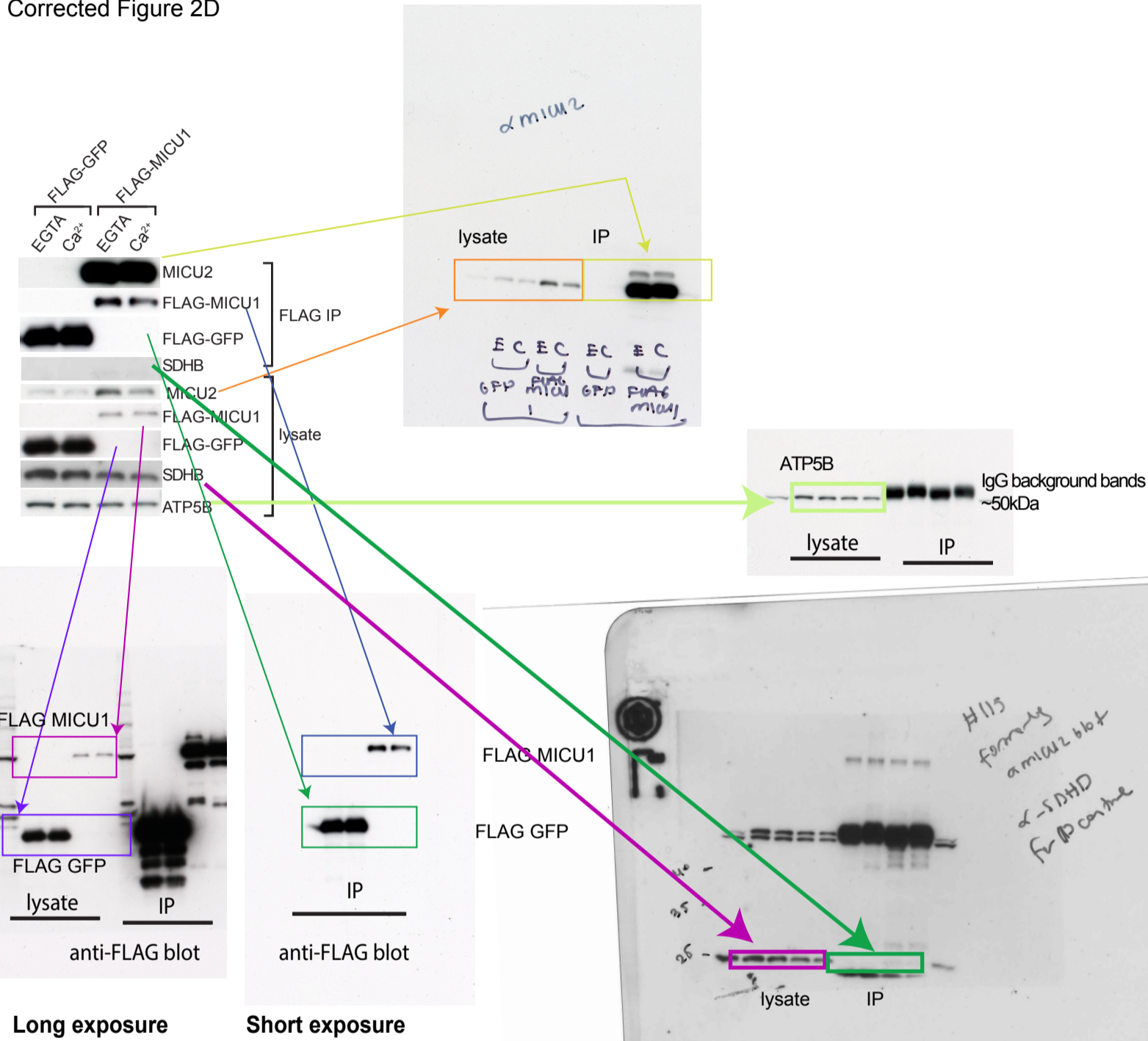

Supplement: S1 File — Annotated scans for all panels in Fig 2D. (PDF) [file pone.0352309.s001.pdf]
